# Injection Injury Caused by Disinfectant During COVID-19: A Case Report

**DOI:** 10.3389/fpubh.2022.851175

**Published:** 2022-04-27

**Authors:** Haifei Shi, Kejiong Liang, Rizwan Ali, Shengquan Xu, Shili Ding

**Affiliations:** ^1^Department of Orthopedics, The First Affiliated Hospital, School of Medicine, Zhejiang University, Hangzhou, China; ^2^Department of Plastic Surgery, The First Affiliated Hospital, School of Medicine, Zhejiang University, Hangzhou, China

**Keywords:** injection injury, COVID-19, occupational safety, debridement, disinfectant

## Abstract

High-pressure injection injury of the hand is a rare but severe emergency, which requires full attention and timely treatment. However, the early symptoms may not be obvious. As the swelling and necrosis progress, the condition gradually worsens, and in severe cases, it may end with amputation. We report a particular case of a hand injection injury, which occurred to a worker who worked overtime to produce disinfectant during the Coronavirus Disease-19 (COVID-19) pandemic. Because of the chemical toxicity of the disinfectant and pressure's damage, although the emergency debridement was promptly performed, we still lost some fingers in the end. In the existing disinfection product manuals, we have not seen any tips on dealing with tissue injection injury. It may reduce workers' attention to injuries, leading to delays in emergency operations.

## Introduction

Hand injection injuries are rare with a not obvious skin lesion. However, at the same time, high pressure and toxicity of the injected fluid may lead to devastating consequences. The swelling and pain may gradually increase, and eventually, the affected fingers had to be amputated owing to ischemic necrosis (1, 2).

In the initial injury, the patient's main complaint is not severe, and the skin wound is small sized; these may cover up the severity of the condition (3). Such injection injuries should promptly receive surgical intervention to debride all ischemic tissue (2). In addition, the possibility of further toxic reactions caused by this fluid gets eliminated in time. Delays in the surgical intervention will increase the chance of permanent hand function loss and finger amputation (4).

Our surgical hand center has 2–4 injection injury treatments every year on average. Most accidents happen on the non-dominant hand during working (5, 6). In the context of the current national fight against Coronavirus Disease-19 (COVID-19), many disinfectant manufacturers are working overtime (7). Through a case of disinfectant injection was injury recently admitted to our center, this article discusses the importance of treating this type of injury quickly and adequately. In addition, the results are compared with previous literature.

## Case Report

**Figure 1:**
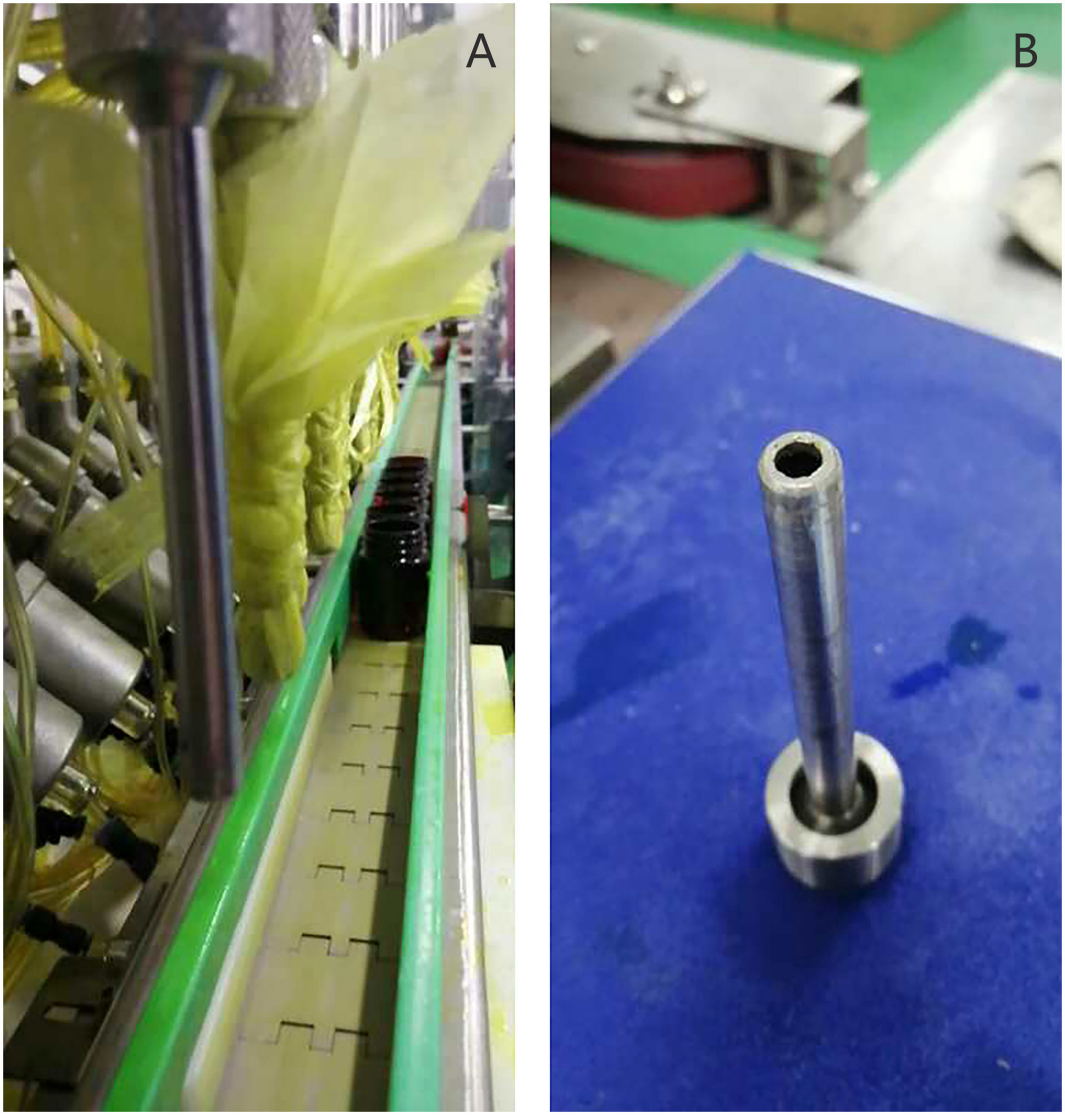
**(A)** Disinfectant filling line. **(B)** Filling head with 5.5 mm inner diameter.

A 39-year-old right-handed female worker suffered from an injection injury to her left hand while operating a disinfectant filling machine. The disinfectant sprayed by the fill head was injected into the finger web between the middle finger and the ring finger of her left hand. The main components of the disinfectant include 1.8–2.2% chlorhexidine gluconate and 63–77% ethanol. The downward force of the filling head is about 500 kg, and the pressure of the filling liquid is ~80 psi (Figure 1).

The patient came to our trauma center 4.5 h after the accident. She complained of noticeable swelling and numbness on the ulnar side of the index finger, middle finger, ring finger, and the radial side of the little finger. At that time, her pain was not severe. All fingers' flexion and extension were restricted because of the swelling. The entry wound on the back of her left hand was about 2-cm long. Unlike the injection injury of other liquids, the adjacent skin of this patient presented ischemic mottled changes. The middle and ring fingers were pale and cold, and the capillary hyperemia reaction was vanished. No fractures or foreign matters were displayed at radiographic imaging, but only significant soft tissue swelling.

## Treatment

**Figure 2:**
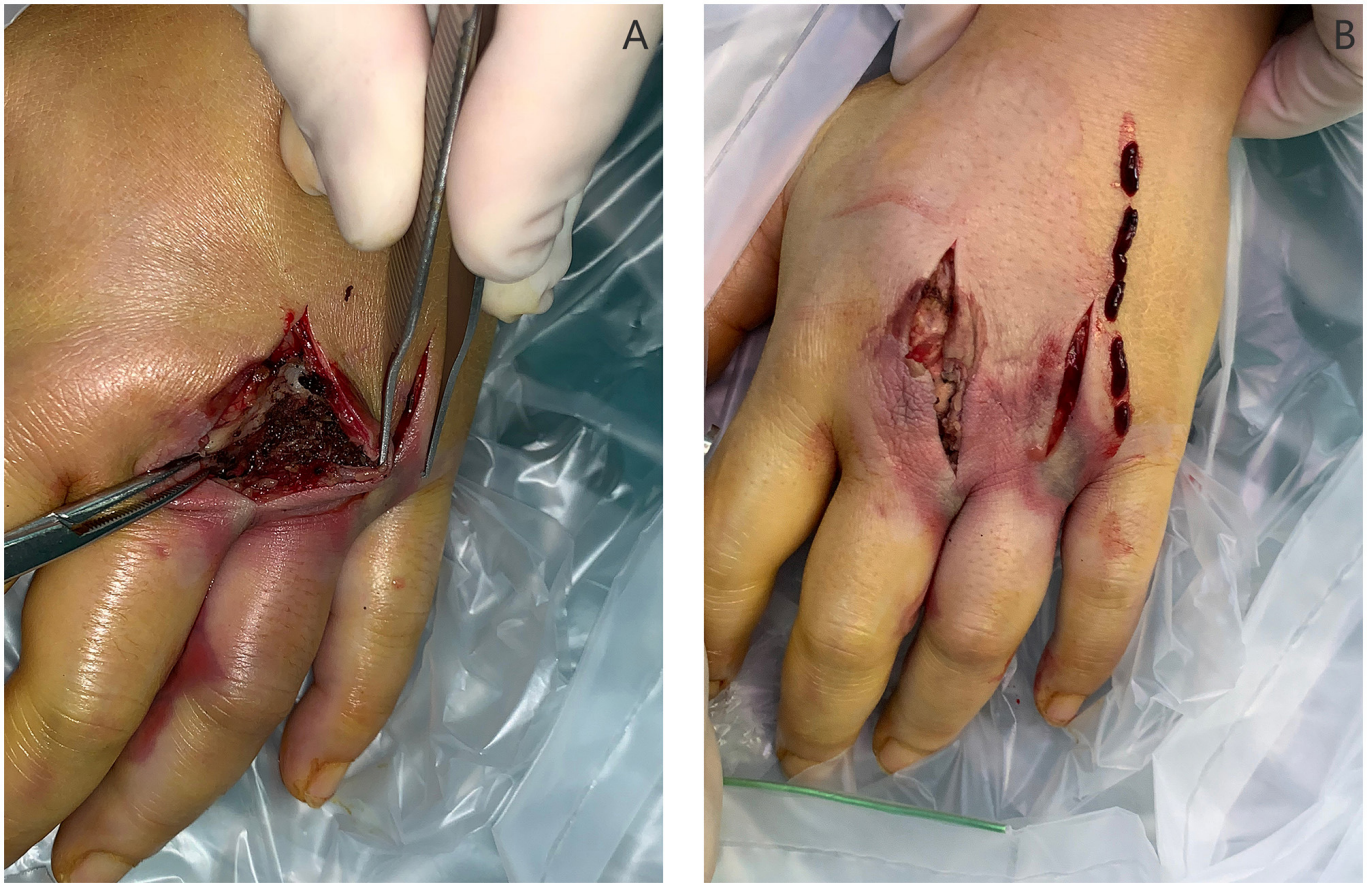
Debridement on the dorsal side: **(A)** Black “carbonized” tissue throughout the entry wound with the pathological report: small pieces of fiber, fat, nerve, and vascular tissue with bleeding. **(B)** Ten centimeters of thrombus removed from the digital dorsal vein.

**Figure 3:**
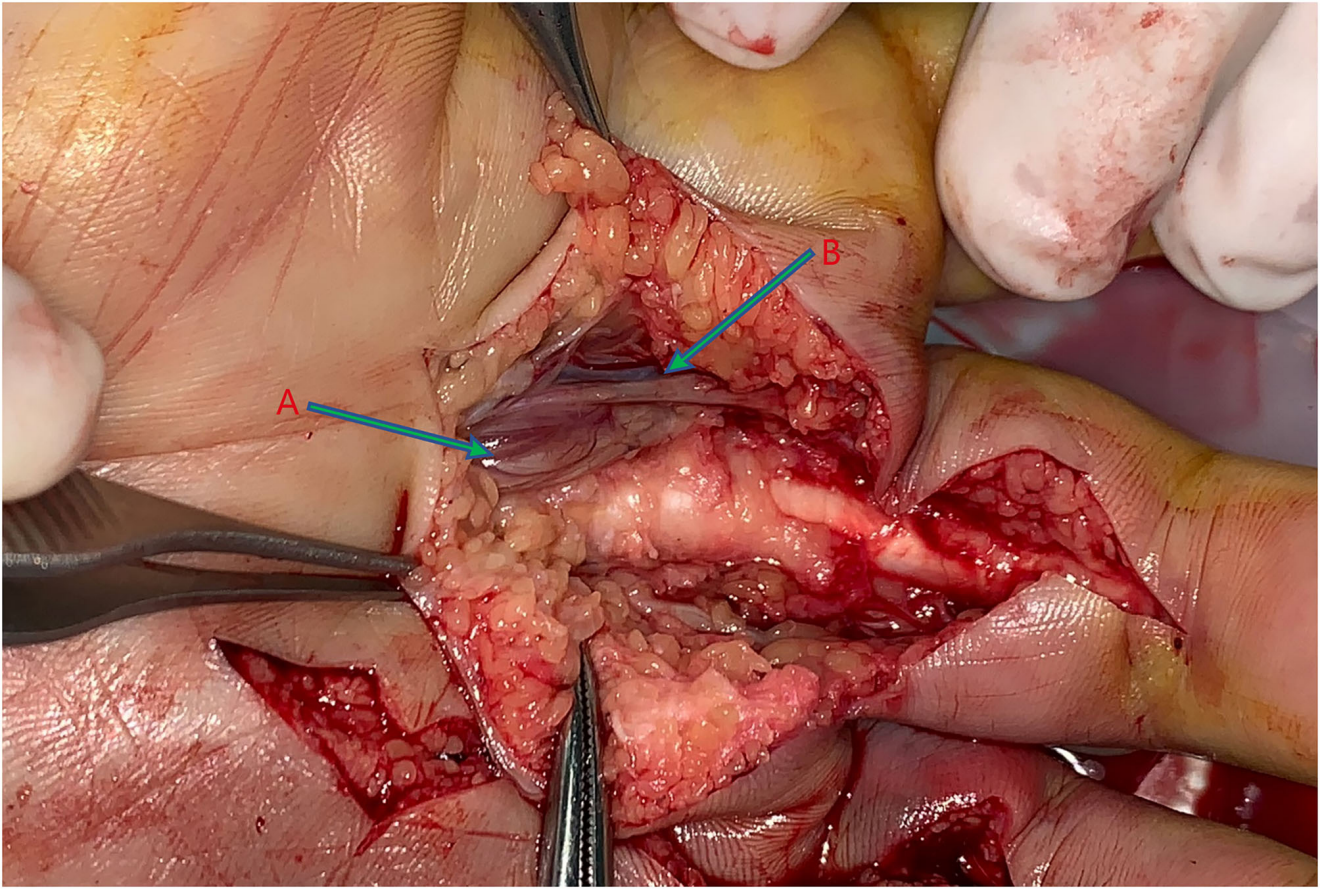
Debridement on the palm side: **(A)** Pallor intrinsic muscles with the pathological report: a small piece of skeletal muscle tissue with purulent inflammation and a small amount of necrosis. **(B)** Neurovascular bundle.

The patient completed an injection of tetanus antitoxin and screening of COVID-19 in the emergency room and underwent 5.5 h of surgery after injury. The left-hand dorsal-side surgical approach is extended longitudinally on the original wound. Additionally, a palm-side approach was made between the third and fourth metacarpal bones. Debridement and decompression were performed in the third and fourth metacarpal spaces. During the operation, a large amount of fluidity was detected in the soft tissue and fascial compartment, and it had a smell of ethanol. Intrinsic muscles and digital nerves in the wound were pale. Intravenous thrombosis was found in the digital dorsal vein, finger arteries, and common palmar digital arteries of the third and fourth fingers (Figures 2, 3). We adopted open thrombectomy to restore the fingers' blood flow. The patient continued on dexamethasone for 3 days. At the same time, the patient received piperacillin/tazobactam intravenously for a duration of 7 days post-operative. After that, she was transitioned to 300 mg of clindamycin, 4 times a day, for another 7 days.

We carefully observed the blood supply of the injured fingers after the debridement. Oxygen saturation and temperature were continuously monitored. On post-trauma day 14, the patient's wound was found to be clean. However, the oxygen saturation of the affected left middle finger and ring finger was on average 9% lower than the healthy side, and the temperature was 4.2°C lower than the healthy side.

**Figure 4:**
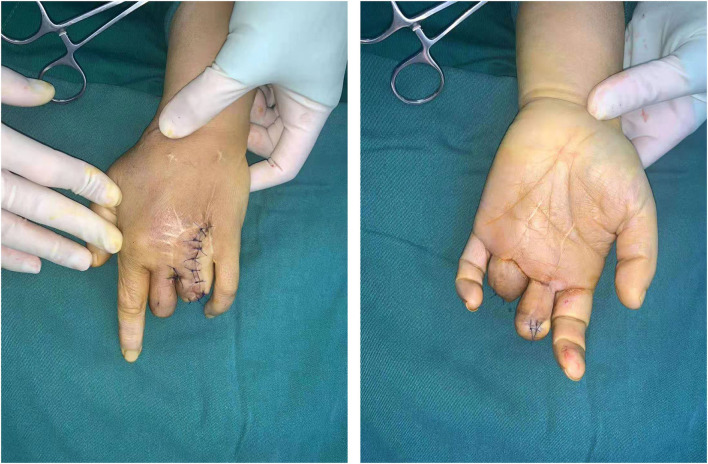
Appearance after flap thinning surgery.

Unfortunately, the injured ring finger and middle finger showed severe necrosis 1 month later. We amputated the necrotic part of the fingers and covered the exposed phalanx at the ring finger with a flap. Then after 8 months, we revised the subcutaneous adipose tissue to convert it into a thin flap (Figure 4).

## Discussion

High-pressure injection injuries usually occur on the index finger, followed by the middle finger and palm (8). The majority of them is manual workers involving compensation of employment injury insurance (9). The injured workers may be unfamiliar with the new equipment or be careless at work.

Most of the time, high-pressure injection injuries appear as innocuous entry wounds and insignificant early symptoms (10). The patient's emergency room visit is often delayed. When they come to the clinic, the presence of the injected material itself and the inflammation it causes often have severely swelled the affected body. Significantly increased tissue pressure can cause vasospasm, resulting in a vicious cycle of swelling and ischemia. Besides, severe fibrosis and inflammation can also increase the risk of amputation (3, 11).

Timely and thoroughly surgical decompression and debridement are the essential factors in improving the prognosis of high-pressure injection injuries (12). Because this is the only factor that medical staff can actively anticipate. Many previous studies do not explicitly mention the exact timing of surgical intervention. For patients injected with toxic substances (paint, diesel, oil, paint thinner, and gasoline), debridement within 6 h can significantly reduce the amputation rate to 40% (3). The most important thing to reduce the preoperative delay time is improving everyone's awareness of the danger of high-pressure perfusion injury.

Early recognition of the severity of the injection injuries is the primary requirement for the treating physician. Radiographs are helpful to identify the material of the injection and determine the extent of the spread.

At the time of injury, many factors can severely impact the prognosis. The first factor is the damage caused by the injection pressure. The initial physical injury can lead to tissue necrosis, neuronal disruption, and vascular damage. It is generally believed that a pressure of 100 psi is sufficient to penetrate the skin (13). The injected fluid preferentially flows along with the neurovascular bundle and fascia. Furthermore, the higher pressure means more fluid injected, more severe the tissue damaged in the end. In this case, the wound on the patient's left hand was caused by the downward force of the filling machine rather than a direct fluid jet.

Different injection sites can withstand different volumes of fluid. It is apparent that the palm has more expansion capacity than the fingers (14). It is another manifestation of the injection pressure on prognosis.

To some extent, this case is unique as compared to other injection injuries. We did not waste much time before surgery, and the injection pressure was not too high, whereas the tissue damage appeared very early and severe. The nature of the injected fluid is another important factor affecting prognosis.

In this case, the components of the injected material were 1.8–2.2% chlorhexidine gluconate and 63–77% ethanol. This is a commonly used and effectively disinfectant. The tissues damage caused by this irritating liquid itself exists obviously. Compared to other common injected substances, such as grease and hydraulic fluid (14–16), this disinfectant is associated with an inferior outcome. Under the chemical toxicity of the liquid, progressive damage may ensue, such as oxidative stress, necrosis, inflammation, ischemia, and apoptotic response. Even after healing, extensive fibrosis around the damaged tissues can severely restrict hand function.

The use of steroids for high-pressure injection injuries remains controversial. For the patients injected with organic solvents, steroids can reduce the inflammation and improve tissue survival.

Last but not least, infection plays a vital role in tissue destruction. Prophylactic antibiotics are recommended for hand injection injuries. Compared with the primary infection, secondary infections caused by ischemia and necrosis are more common (17). A combination of antimicrobial agents covering the gram-positive cocci and gram-negative rod should be chosen as empiric antibiotic treatment.

## Important Lesson

Safe operation is always the most important principle at work. This patient was injured on the 1st day after the Spring Festival holiday. Because it happened in the particular period during which COVID-19 just outbreak, only a part of mask factories, disinfectant factories, and other essential factories recalled workers to resume production. From the patient's perspective, she worked overtime in a specific state of mind. During the hospital stay, this patient repeatedly told us that she should not be so careless. Because her wound was only 2 cm, and the pain was not apparent initially. In the early post-operative period, she was anxious and unacceptable about the severity of the injury and the possibility of amputation. After our repeated communication and comfort, she actively cooperated with subsequent treatment and rehabilitation. As this case stands, we think that psychological intervention, when necessary is also an important measure to ensure safe production.

We checked every instruction on this kind of disinfectant, which can find on the market. The instructions list first aid measures after swallowing, eye contact, and so on, but no mention of injection injuries. High-pressure injection injuries should receive more attention, such as the formulation of instructions and safety education in factories need to be strengthened.

The clinician should not be misled by small skin lesions caused by injection injuries, and even the initial clinical presentations are not severe. In order to avoid the severe decrease in hand function, it is necessary to perform extensive and complete debridement immediately, moreover, anti-infective treatment timely after the operation.

## Data Availability Statement

The original contributions presented in the study are included in the article/Supplementary Material, further inquiries can be directed to the corresponding authors.

## Ethics Statement

The studies involving human participants were reviewed and approved by Clinical Research Ethics Committee of the First Affiliated Hospital, College of Medicine, Zhejiang University. The patients/participants provided their written informed consent to participate in this study. Written informed consent was obtained from the individual(s) for the publication of any potentially identifiable images or data included in this article.

## Author Contributions

HS: conceptualization, methodology, and writing—original draft. KL: data curation, writing—original draft preparation, and writing—review and editing. RA and SD: writing—review and editing. SX: visualization and investigation. All authors contributed to the article and approved the submitted version.

## Conflict of Interest

The authors declare that the research was conducted in the absence of any commercial or financial relationships that could be construed as a potential conflict of interest.

## Publisher's Note

All claims expressed in this article are solely those of the authors and do not necessarily represent those of their affiliated organizations, or those of the publisher, the editors and the reviewers. Any product that may be evaluated in this article, or claim that may be made by its manufacturer, is not guaranteed or endorsed by the publisher.
