# Supplementary figures and images for: Injection Injury Caused by Disinfectant During COVID-19: A Case Report
Source: Front Public Health. 2022 Apr 27;10:851175. doi: 10.3389/fpubh.2022.851175 (PMC9092298; doi:10.3389/fpubh.2022.851175)

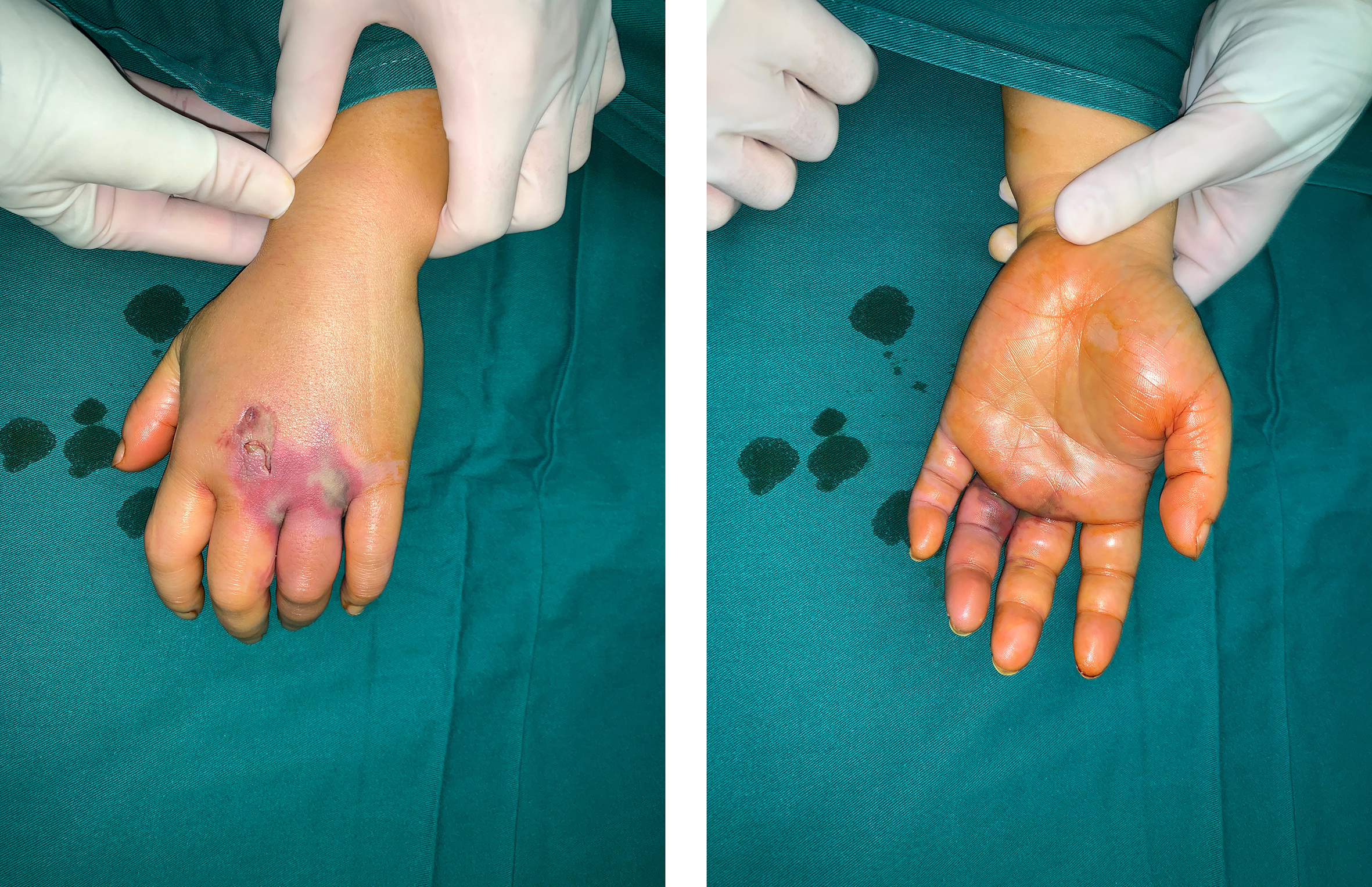

Supplement: Supplementary file 1 [file Image_1.JPEG]
